# Intraprosthetic Dislocation Following Dual-Mobility Total Hip Arthroplasty: A Case Report

**DOI:** 10.7759/cureus.107069

**Published:** 2026-04-14

**Authors:** Abhinandan S Punit, Akshay Nayak, Yathisha SR, Yeshwanth M Reddy, Abin M Nizar

**Affiliations:** 1 Orthopaedic Surgery, Narayana Health City Hospital, Bengaluru, IND

**Keywords:** dual-mobility total hip arthroplasty, hip instability, implant failure, intraprosthetic dislocation, polyethylene liner dislocation, revision hip arthroplasty

## Abstract

Dual-mobility total hip arthroplasty has been increasingly adopted to improve joint stability and reduce postoperative dislocation. Despite these advantages, this implant design is associated with specific modes of failure. One such complication is intraprosthetic dislocation, which occurs when the femoral head disengages from the polyethylene liner. Although uncommon, this condition is clinically important and may not be readily identified at initial presentation.

We report a case involving a 67-year-old woman who presented with sudden onset of hip pain and mechanical restriction of movement two years after undergoing dual-mobility total hip arthroplasty for a fracture neck of femur. Radiographic assessment revealed abnormal positioning of the femoral head within the acetabular component, suggesting dissociation of the polyethylene liner. Computed tomography further supported this diagnosis, and inflammatory markers were within normal limits. Surgical exploration confirmed intraprosthetic dislocation of the liner, while both the femoral stem and acetabular shell were well fixed. Management consisted of removing the mobile bearing and converting the articulation to a fixed polyethylene liner within the existing acetabular cup. Postoperatively, the patient recovered without complications and demonstrated sustained functional improvement without recurrent instability at one year.

This case underscores that intraprosthetic dislocation remains a potential cause of mechanical failure even with contemporary dual-mobility implants. Because clinical symptoms may be subtle, careful attention to characteristic imaging findings is essential to avoid diagnostic delay. When implant fixation is preserved, selective exchange of modular components can provide a successful and less invasive treatment option. Prompt recognition and appropriate surgical intervention are crucial to prevent further prosthetic damage and to achieve satisfactory functional outcomes.

## Introduction

Instability continues to be an important complication after primary total hip arthroplasty (THA), despite ongoing improvements in implant design and surgical technique. Reported dislocation rates at one year range from 2% to 10% [1]. To help reduce this risk, dual-mobility (DM) THA has become widely used. In this design, a small femoral head moves within a polyethylene liner, which then articulates within a metal acetabular shell, providing greater stability compared with conventional implants. Studies have shown that this design is associated with lower dislocation rates [2].

However, DM implants also have their own specific complications. One such complication is intraprosthetic dislocation (IPD), where the femoral head separates from the polyethylene liner. Although uncommon, it is a distinct failure mode that can be easily overlooked in routine practice and often requires revision surgery. The present case report describes a case of IPD following DMTHA and highlights the importance of recognising it early and managing it appropriately.

## Case presentation

A 67-year-old woman presented to the outpatient department with a two-week history of sudden-onset left hip pain and mechanical locking. She had undergone a left THA two years earlier for a fracture neck of femur following trauma. Her postoperative course after left THA had been complicated by delayed wound healing, which was treated with vacuum-assisted closure (VAC) therapy, after which recovery was uneventful. Her current symptoms began abruptly two weeks prior to presentation, after she heard an audible “cluck” while sitting on a toilet seat, followed immediately by pain and difficulty in moving the hip. Since then, she experienced increasing difficulty with routine activities of daily living, including walking, climbing stairs, and performing basic movements such as sitting down and rising from a bed or toilet seat.

Her medical history included benign paroxysmal positional vertigo, hypertension, and type 2 diabetes mellitus. She had a body mass index (BMI) of 38.2 kg/m². On examination, localized tenderness was noted over the anterior joint line and greater trochanter of the left hip. Hip movements were restricted, particularly flexion, abduction, and rotational movements. There were no neurovascular deficits or signs of generalized lymphadenopathy.

**Figure 1:**
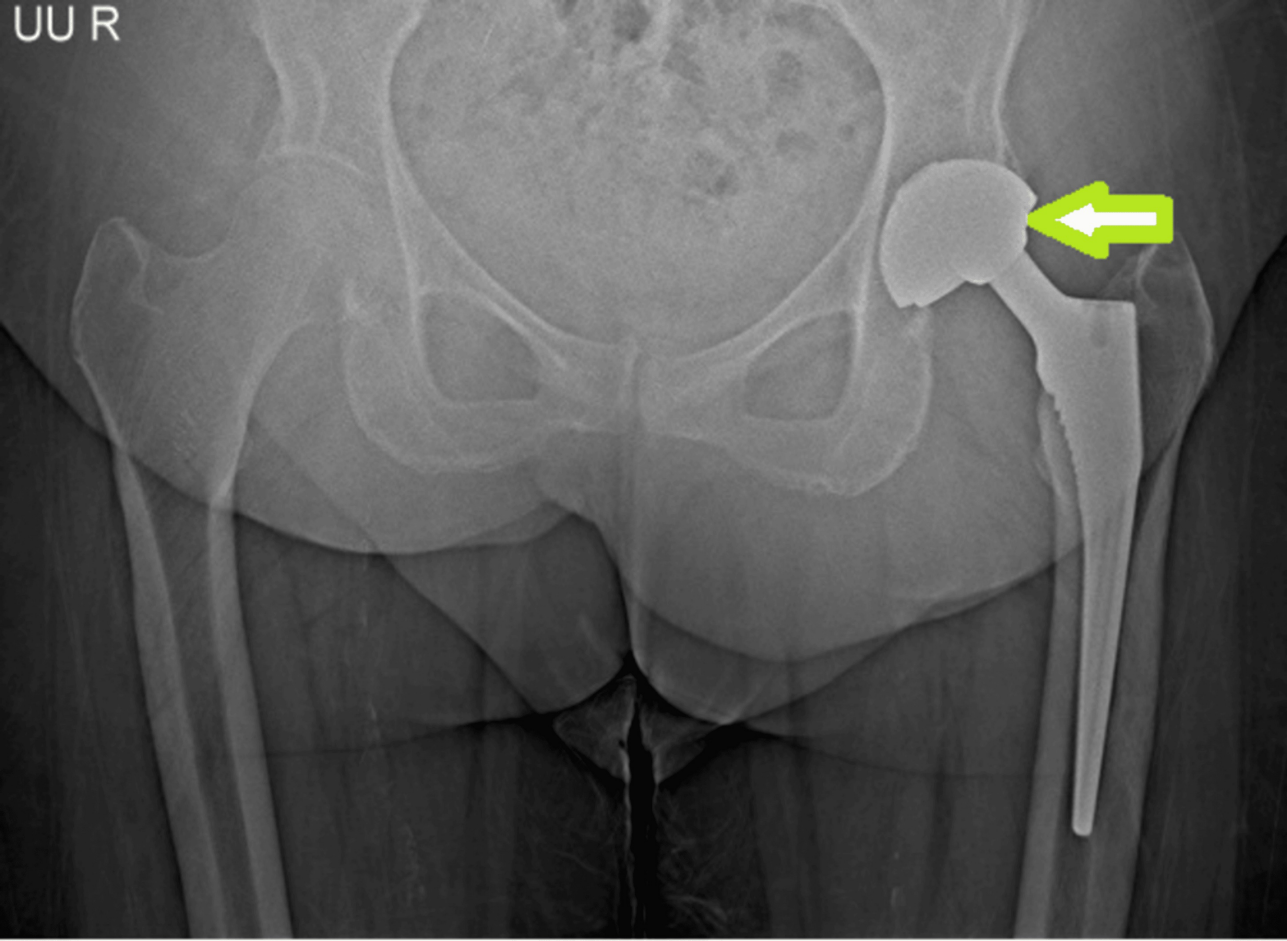
Radiographic imaging of the left hip postoperatively (two years after total hip arthroplasty). Anteroposterior (AP) view showing the position of the femoral component and acetabular shell. The green arrow demonstrates eccentric positioning of the femoral head within the acetabular component.

**Figure 2:**
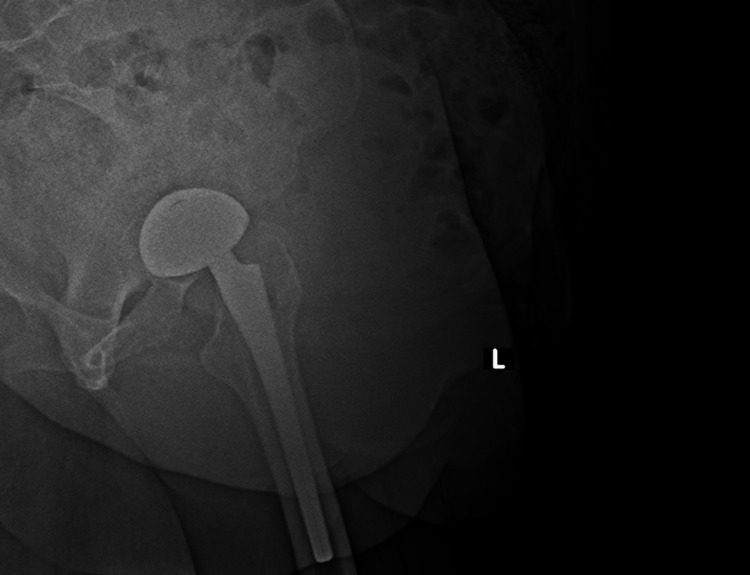
The lateral radiograph of the left hip joint (two years after left total hip arthroplasty) showing displacement of femoral head prosthesis with respect to the acetabular cup.

**Figure 3:**
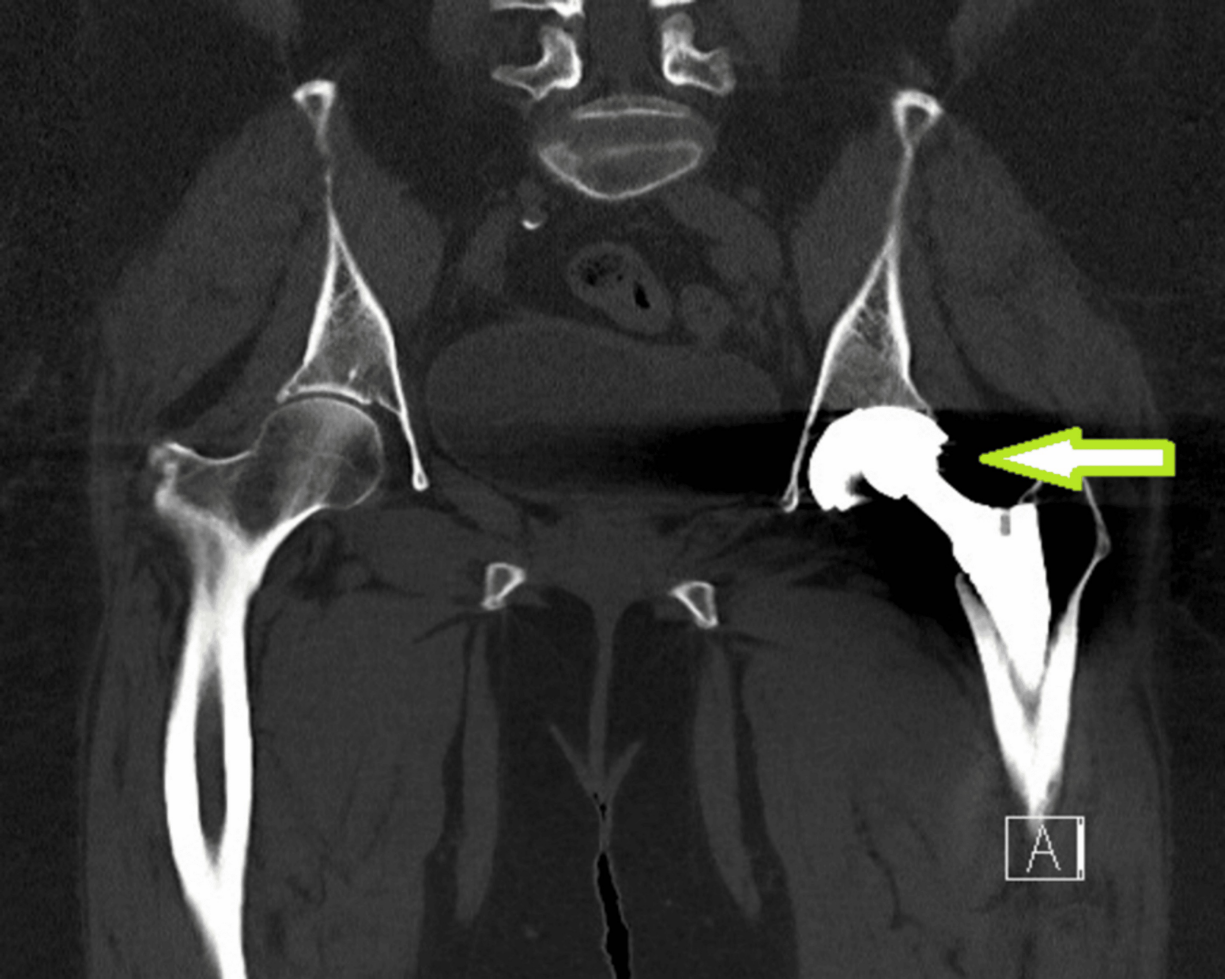
Pre-operative computed tomography scan coronal section of pelvis with both hip joints. Green arrow pointing at the eccentric and lateral position of left femoral head component within the acetabular shell.

Plain radiographs (anteroposterior pelvis and lateral view of the left hip) demonstrated lateral displacement of the femoral head relative to the acetabular component (Figures 1, 2) without evidence of loosening. Computed tomography showed eccentric and lateral positioning of the femoral head, raising suspicion of polyethylene liner dissociation (Figure 3). Laboratory investigations, including complete blood count (hemoglobin: 12g/dL, total leukocyte count: 7400/cu mm, platelet: 2.32lakhs/cu mm), erythrocyte sedimentation rate (9mm/hour), and C-reactive protein (0.8mg/dL), were within normal limits.

**Figure 4:**
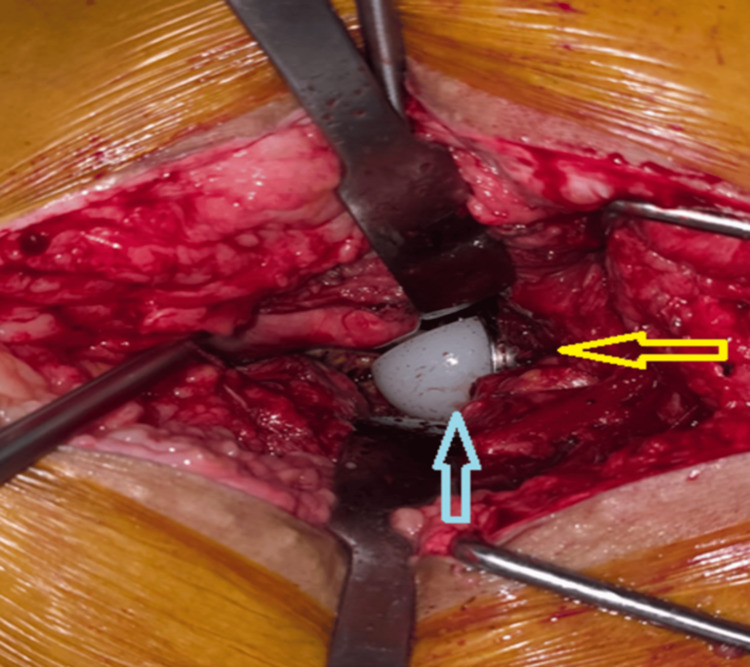
Intra operative image showing the left side femoral head and polyethylene liner. The femoral head was dislocated from the liner, confirming the diagnosis of intraprosthetic dislocation. Yellow arrow depicting normal position of acetabular shell. Blue arrow depicting dislocated polyethylene liner component.

A trial of closed reduction for the IPD was attempted on the operating table; however, the hip continued to redislocate. As a result, an open reduction was then performed. Unfortunately, despite this effort, the hip remained unstable. Given the patient’s symptoms and functional demands, revision hip arthroplasty was planned. Surgery was performed under general anesthesia with the patient in the right lateral decubitus position. The previous surgical incision was utilized, and dissection was carried out in layers through the subcutaneous tissue, deep fascia, and iliotibial band. Intraoperatively, there was no evidence of purulence, metallosis, or infection. IPD of the polyethylene liner was identified and removed (Figure 4). The femoral stem and acetabular shell were found to be well fixed.

Trial components were inserted to assess stability. Based on intraoperative findings, the articulation was converted from a DM construct to a metal-on-polyethylene articulation using a fixed polyethylene liner within the existing uncemented acetabular shell. A 10° oblique polyethylene liner (28 mm) and a modular metal femoral head (MB/28) were implanted, and the hip was subsequently reduced. Stability was confirmed through a full range of motion. The wound was thoroughly irrigated and closed in layers over a drain.

**Figure 5:**
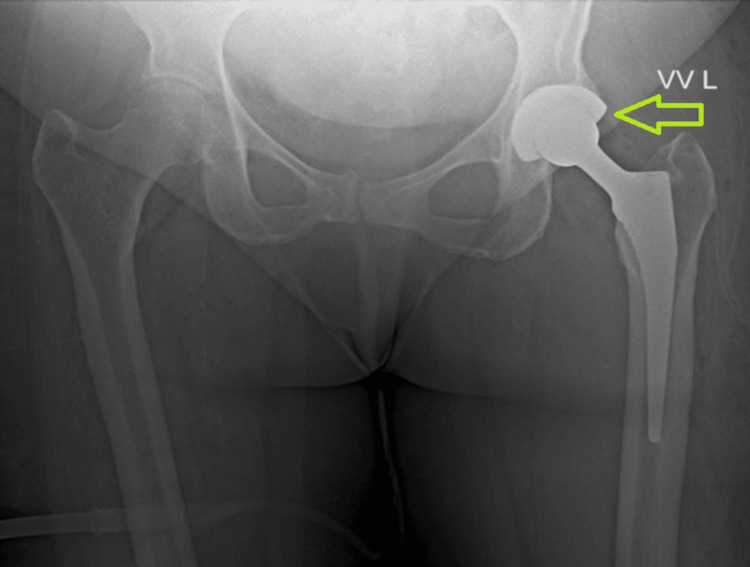
Postoperative radiograph after component revision. Anteroposterior plain radiograph of pelvis with both hip joints showing the newly implanted components, including the modular femoral head and polyethylene liner. The components are properly aligned, with no signs of dislocation. Green arrow showing well reduced left hip joint.

The postoperative period was uneventful. Radiographs confirmed appropriate positioning of the femoral head within the acetabular component (Figure 5). Wound inspections on postoperative days two and five showed satisfactory healing. The patient was discharged on postoperative day five with instructions to continue hip mobilization exercises. Sutures were removed on postoperative day 16 after complete wound healing.

At follow-up visits at six weeks, three months, six months, and one year, the patient demonstrated progressive improvements in hip motion and functional mobility, with no recurrence of instability or mechanical symptoms.

## Discussion

DM implants have been used in THA since their introduction in the 1970s [3]. These systems are broadly categorized into monoblock (anatomic) and modular designs [4]. Monoblock implants consist of a press-fit acetabular component articulating with a mobile polyethylene liner that captures a femoral head. Modular systems comprise a press-fit acetabular shell that accepts a cobalt-chromium liner, which serves as the articulating surface for the polyethylene liner enclosing the femoral head.

An advantage of the modular design is the flexibility it provides in both primary and revision settings, as liner removal allows access for screw fixation through the acetabular shell. Several studies have demonstrated that DM bearings reduce dislocation rates after both primary and revision THA [1,2]. Additional reported benefits include increased jump distance, favorable wear characteristics, and a greater range of motion [5,6]. Current evidence suggests that neither monoblock nor modular designs are superior in terms of dislocation rates [4]. The DM concept combines a small articulation to limit wear with a larger effective articulation to enhance stability.

Despite these advantages, IPD remains a rare but specific complication of DM implants [5,7]. IPD refers to separation of the femoral head from the polyethylene liner, with subsequent articulation of the femoral head directly against the metal-backed shell. This is typically related to failure or wear of the retentive rim that normally constrains the femoral head within the liner.

In earlier generations of DM implants, polyethylene wear was the primary mechanism leading to IPD. Contributing factors included lower-quality polyethylene and unfavorable femoral neck designs, such as skirted or rough-surfaced necks, which accelerated liner wear [8]. Rim wear may also result from repetitive impingement between the femoral neck and liner, particularly in designs with large neck diameters or inadequate impingement clearance. Reported rates of IPD in early designs ranged from 0.7% to 5.2% [5,7].

With the introduction of highly cross-linked polyethylene in modern DM designs, the incidence of IPD has decreased substantially. Epinette et al. reported no cases of IPD at five-year follow-up in a prospective series of patients treated with contemporary DM constructs [9]. Similarly, a systematic review found that multiple studies with follow-up exceeding 10 years reported no cases of IPD, suggesting improved implant performance and longevity [6].

Nevertheless, IPD remains a clinically relevant complication. In modern implants, early IPD is more commonly related to iatrogenic factors rather than polyethylene wear. De Martino et al. demonstrated that many early cases were associated with attempted closed reduction of a dislocated DM hip [7]. This mechanism has been described as the “bottle-opener effect,” in which the polyethylene liner engages against the acetabular rim or pelvic bone during reduction maneuvers. Addona et al. reported a high proportion of IPD cases occurring after closed reduction attempts [10]. Similar findings were described by Rhyu et al., who reported dissociation of the polyethylene liner following closed reduction of a dislocated DM hip [11], highlighting the importance of careful reduction techniques and post-reduction radiographic evaluation.

Delayed diagnosis of IPD may result in damage to the acetabular component, soft tissue metallosis, and abnormal metal ion levels [12]. To improve detection, imaging features such as eccentric femoral head positioning and subtle liner displacement should prompt further evaluation. Radiographic markers within the polyethylene liner have also been proposed to facilitate diagnosis in ambiguous cases [13,14].

Additional mechanisms of IPD include failure of the liner-head retention mechanism, improper seating of the liner at implantation, and technical factors during closed reduction [12,15]. Anatomical considerations, such as small acetabular component size, may reduce jump distance and stability, increasing susceptibility to disengagement [16]. Obesity is a recognized risk factor for instability following THA. Although DM implants generally provide improved stability in obese patients, increased mechanical loading and altered biomechanics may contribute to early failure, including IPD, in selected cases [17]. While DM or constrained liners have been shown to reduce dislocation risk in obese patients, as reported by Philippe Hermigou et al. [18], these constructs may still be susceptible to mechanical complications under increased load.

Recent contemporary series confirm that IPD can occur even with modern DM constructs. Mallett et al. reported the incidence, management, and outcomes of IPD in a large institutional cohort and demonstrated that femoral head dissociation may occur despite advances in implant design and polyethylene technology [19]. Identified risk factors included previous instability, repeated or forceful closed reduction attempts, and mechanical impingement between the femoral neck and polyethylene liner.

In the present case, IPD occurred within two years of DMTHA, highlighting the combined influence of implant-related and patient-related factors. Contemporary series report IPD rates ranging from 0% to 0.3% in modern constructs [12,20].

## Conclusions

DMTHA is an effective strategy for reducing postoperative instability; however, IPD is a rare but important implant-specific complication. This condition may present with subtle symptoms and can easily be overlooked if not considered in the differential diagnosis.

Surgeons should maintain a high index of suspicion in patients presenting with pain or mechanical symptoms following DMTHA. Early diagnosis through appropriate radiographic evaluation, particularly anteroposterior and lateral views showing eccentric positioning of the femoral head, is crucial for timely management. When the femoral stem and acetabular cup remain well-fixed, isolated modular component exchange can be a reliable and less invasive treatment option. Prompt recognition and intervention can prevent further implant damage and improve functional outcomes. While closed reduction should be attempted first in the operating theatre, if unsuccessful, open reduction is necessary. Persistent instability requires component revision, as demonstrated in this case.
